# The possible role of Dickkopf-1, Golgi protein- 73 and Midkine as predictors of hepatocarcinogenesis: a review and an Egyptian study

**DOI:** 10.1038/s41598-020-62051-6

**Published:** 2020-03-20

**Authors:** Abdel-Rahman N Zekri, Mohamed EL Kassas, El SayedTarekAbd El Salam, Reem Mostafa Hassan, Marwa Mohanad, Reham Mohamed Gabr, Mai M. Lotfy, Rania A. Talaat Abdel-zaher, Abeer A. Bahnassy, Ola S. Ahmed

**Affiliations:** 1 Molecular Virology and Immunology Unit, Cancer Biology Department, National Cancer Institute, Cairo University, Cairo, Egypt; 2 Endemic Medicine Department, Faculty of Medicine, Helwan University, Cairo, Egypt; 3 Botany and Microbiology Department, Faculty of Science, Cairo University, Cairo, Egypt; 4 Clinical and Chemical Pathology Department, Faculty of Medicine, Cairo University, Cairo, Egypt; 5 Biochemistry Department, College of Pharmaceutical Sciences and Drug Manufacturing, Misr University for Science and Technology, Giza, Egypt; 6 Molecular pathology unit, pathology Department, National Cancer Institute, Cairo University, Cairo, Egypt

**Keywords:** Tumour biomarkers, Diagnostic markers, Predictive markers

## Abstract

Hepatocellular carcinoma (HCC) is the second most common cause of cancer-related death worldwide. The use of alpha fetoprotein (AFP) alone was not an accurate biomarker for HCC despite its high specificity. Therefore, we assessed the possible role of serum biomarkers that have been mentioned briefly in previous studies on Egyptian patients ion top of HCV. However these studies included small number of patients and did not assess the different stages of hepatocarcinogenesis. In the current study we assessed 1) the expression levels of Golgi protein 37(GP73),Midkine (MDK) and Dickkopf-1(DKK-1) proteins separately and in combination at different stages of hepatocarcinogenesis. GP73, MDK and DKK-1 proteins were assessed in 238 individuals divided into 4 groups (HCC, chronic HCV, and chronic HCV with cirrhosis and healthy subjects as a control) Serum levels of GP73, MDK, and DKK-1 were assessed in all subjects by ELISA. Serum levels of the studied markers were significantly higher in HCC compared to other groups (p < 0.001). The ROC curve analysis for the studied markers showed 1) 88.5% sensitivity, 80.6% specificity, 69% PPV, 93.5% NPV and (AUC 0.91)for MDK; 2) 93.6%, 86.9%, 77.7%, 96.5% for DKK-1. 3) 91%, 85%, 74.7%, 95% (AUC 0.96) for GP73 and 4) 74.4%, 84.4%, 69.9%, 87.1% (AUC 0.81) for AFP. Serum levels of GP73, MDK, and DKK-1 are comparable to AFP as promising predictor biomarkers for HCC patients from Egypt. A two markers panel including Gp73 and DKK-1 showed the highest specificity and sensitivity among different markers combinations.

## Introduction

Hepatocellular carcinoma (HCC) is the second most common cause of cancer-related death worldwide and it was responsible for nearly 746,000 deaths in 2012^1^. Hepatitis C virus (HCV) infection has been considered the second most common risk factor of HCC^2^. Recent screening programs and the modern, highly sensitive imaging techniques used now such as ultrasound, CT and magnetic resonance imaging (MRI) have greatly improved early detection of HCC, which impacted positively on patients’ outcome, mainly response to treatment and survival rates^3^.

Serum biomarkers have also been used as a tool to screen and/or diagnose HCC at an early stage of the disease in several recent studies. Alpha-fetoprotein(AFP) is one of the thoroughly investigated biomarkers in different solid tumors, premalignant lesions or inflammatory conditions. It is a glycoprotein which is synthesized in large amounts by the yolk sac and liver during embryonic development. It disappears gradually after birth and is it only re-expressed in HCC. Therefore, it has been widely used clinically as a tumor marker for HCC^4^. The sensitivity and specificity for AFP varied widely in different studies at the early stages of hepatocarcinogenesis^5^. Therefore, identification of more reliable serum biomarkers; with high sensitivity and specificity; which complement AFP and improves clinical outcomes of those patients are highly required.

Recent studies have shown that other proteins could also be used as sensitive and specific markers for hepatitis as well as for HCC including the Golgi protein 73 (GP73); which is also named Golgi phosphoprotein 2 “GOLPH2, the Dickkopf-1(DKKpf-1 or DKK-1), and Midkine (MDK). These biomarkers have been used as potential, accurate, simple and non-invasive biomarkers for early detection of HCC^6–8^.

The GP73 is an occupant Golgi-specific membrane protein which is expressed by biliary epithelial cells in the normal liver. It increases in hepatitis, liver cirrhosis and maximally in HCC and it also accompanies the fibro-genesis stage (Maitra and Thuluvath, 2004)^9^. However, some studies demonstrated that GP73 serum level in HCC patients are markedly overlapped by the presence of cirrhosis, which affects its diagnostic accuracy^10^.

DKK-1 is a secretary protein with 266-amino acid (35 kDa) which is secreted directly into the blood stream. It is minimally expressed in the normal human adult tissues except the placenta and embryonic tissues. DDK1 increases in patients with various types of cancers including HCC, and therefore it could be used as a novel prognostic indicator in HCV- infected patients and it may also be used for early detection of HCC^11^.

Midkine (MDK) is a member of the heparin-restricting developmental factor family of proteins which was recognized as a cytokine in the HCV- associated HCC patients. It is expressed in several human malignancies including HCC among others. Some reports showed that the serum level of MDK is usually raised in most HCC cases, and that it may have an important role in AFP-negative patients as well as in early stage tumors^12,13^. Based on this data we sought to assess the possible diagnostic and prognostic roles of GP73, MDK, DKK proteins in the early detection of HCC in chronic HCV infected patients from Egypt.

## Methods

### Study design

This case control study included 238 participants who were admitted to the clinics of the National research center (NRCH) and the National Cancer Institute (NCI), Cairo University during the period from November 2014 to October 2017.

The study protocol was approved by the institutional review board of the National Hepatology and Tropical Medicine Research Institute (NHTMRI) and the National Cancer Institute (NCI). The organization No. is IORG0003381 and the IRB NO· is IRB00004025). All steps of the research was performed in accordance with relevant guidelines (according to 2011 declaration of Helsinki). A written informed consent was obtained from each patient and control subject prior to enrollment in the study. AllHCC patients were diagnosed according to the American Association for the Study of Liver Diseases (AASLD) Practice Guidelines^14^ and staging was done based on Barcelona Clinic Liver Cancer (BCLC) staging system^15^.

Serum biomarkers in HCC patients were compared to chronic HCV patients either with or without liver cirrhosis as well as with apparently healthy donors as a control. All healthy individuals showed normal abdominal ultrasonography and no clinical or biochemical evidence of liver disease. HCV infection was confirmed in all cases by PCR using Artus HCV RT-PCR, QIAGEN PCR detection kit (Applied Bio systems 7500 fast system, ThermoFisher Scientific, USA) to detect HCV RNA expression levels. All patients were subjected to complete liver profile tests [including serum albumin, prothrombin time, bilirubin, and transaminases (ALT and AST)], complete blood picture (CBC), serum creatinine and Alfa fetoprotein using the automated biochemical Cobas 8000 modular analyzer c701/702, Roche, USA)]. Imaging studies included routine abdominal ultrasonography for in all subjects, and triphasic CT or dynamic MRI in patients with HCC. The exclusion criteria included patients with other liver diseases such as HBV co-infection and patients with a previous exposure to HCV antiviral therapy.

### Sample preparation and measurement of serum biomarkers

Blood samples were collected from HCC patients at the time of diagnosis prior to surgery or any other treatment modalities. All serum samples were centrifuged, aliquoted and stored at −80 °C until being used for testing.

Assessment of the studied markers for all subjects was done using ELISA based kits (CLOUD CLONE CORP, Houstory, USA) according to manufacturers’ instructions with optical density (O.D) measured at 450 nm wave length in a micro-plate reader (TECAN-Absorbance Reader INFNIT F50, Austria). The concentration of each protein was calculated according to manufacturers’ instructions as follows;GP73 (0.625–40 ng/ml), MDK (15.625–1000 pg./ml), and DKK-1 (31.25–2000 pg/ml).

### Statistical analysis

The SPSS, version 22.0 (IBM SPSS, Armonk, NY, USA) and graph-pad prism 7 were used for statistical analyses. The continuous variables were compared with one-way ANOVA with post-hoc Tukey test and Mann–Whitney U test was used to differentiate between the level of studied markers for each independent group. Chi-square was used for gender comparison among studied groups. The Receiver Operating Characteristic (ROC) curves were performed and Area under the curve (AUC) with 95% CI was calculated to compare the diagnostic ability of each marker. Pearson’s Chi-square correlation was used to assess the association between studied biomarkers and tumor size. Logistic regression model was used to assess the diagnostic performance of studied markers. All p-values are two-tailed and P < 0.05 was considered to indicate a statistically significant difference.

## Results

### Patient characteristics

A total of 238 participants were recruited in this study, including 78 HCV- related HCC patients, 40 patients with HCV-related liver cirrhosis, and 40 chronic HCV patients without cirrhosis. Also included are 80 healthy individuals with no history of liver disease or alcohol consumption as a control group. The mean age for the studied participants was 48.5 ± 14.7 years. One hundred and fifty-fourpatients (64.7%) were males and eighty-four (35.3%) were females. The male to female ratio was (1.8: 1). Clinical features of the studied groups are shown in (Table 1).

**Table 1:** The clinical feature of the studied groups.

	HCC (n = 78)	Cirrhotic (n = 40)	Non-cirrhotic (n = 40)	Control (n = 80)	P* value
**Age**					<0.001
mean ± SD	58.27 ± 9.7^ab^	58.2 ± 7.06^ab^	50.7 ± 10.25	40.1 ± 9.6	
(range)	(27–74)	(40–69)	(29–69)	(31–64)	
**Gender**					<0.001
Male	46 (59)	26 (65)	23 (57.5)	59 (73.8)	
Female	32 (41)	14 (35)	17 (42.5)	21 (26.2)	
**ALT**					<0.001
Mean ± SD	68.36 ± 34.1^a^	80.37 ± 57.8^a^	42.9 ± 20.5^a^	22.9 ± 6.0	
(range)	(12–140)	(18–242)	(14–330)	(6–25)	
Median	50.5	50	42.5	12	
**AST**					<0.001
Mean ± SD	56.96 ± 26.6^ab^	66.5 ± 46.9^ab^	57.2 ± 53.5^a^	13 ± 4.2	
(range)	(9–191)	(19–335)	(19–110)	(12–40)	
Median	63.5	63.0	40.5	23.0	
**Albumin**					<0.001
Mean ± SD	3.4 ± 0.53^ab^	3.5 ± 0.5^ab^	4.0 ± 0.68	3.99 ± 0.64	
(range)	(2.1–4.5)	(2.8–4.7)	(1.4–5.3)	(1.2–4.9)	
Median	3.4	3.6	4.0	4.1	
**AFP**					<0.001
Mean ± SD	29.5 ± 27.1^abc^	13.6 ± 11.99	7.2 ± 4.7	5.6 ± 1.9	
(range)	(1.4–100.0)	(1.0–64.0)	(10–20)	(2.6–9.6)	
Median	18.9	10.0	6.0	5.5	
**Hb**					<0.001
Mean ± SD	12.34 ± 1.6^ab^	12.9 ± 1.8^a^	13.7 ± 5.8	14.2 ± 1.3	
(range)	(9.1–15.5)	(10.1–17.5)	(11.4–16.4)	(12.5–16.5)	
Median	12.6	13.0	13.6	13.9	
**WBCs**					<0.001
Mean ± SD	5.4 ± 2.1^a^	5.3 ± 1.7^a^	5.8 ± 2.1^a^	6.9 ± 1.4	
(range)	(1.8–11.1)	(2.6–9.3)	(5.0–11.0)	(4.2–10.0)	
Median	5.7	5.0	5.5	6.7	
**Platelets**					<0.001
Mean ± SD	127.4 ± 62.8^ab^	120.45 ± 41.2^ab^	216.3 ± 55.5^a^	312 ± 85.1	
(range)	(3.8–308)	(51.0–224.0)	(88.0–356.0)	(3.8–435.0)	
Median	107.5	115.5	204.5	310.5	

*ANOVA statistics, post-hoc tukey

Gender wascompared using Chi-Square test.

^a–c^ Groups bearing different initials are significantly different. ALT: Alanine aminotransferase; AST: Aspartate aminotransferase; AFP: α fetoprotein; Hb: hemoglobin; WBCs: White blood cells.

### The levels of serum biomarker in relation to tumor size

**Figure 1:**
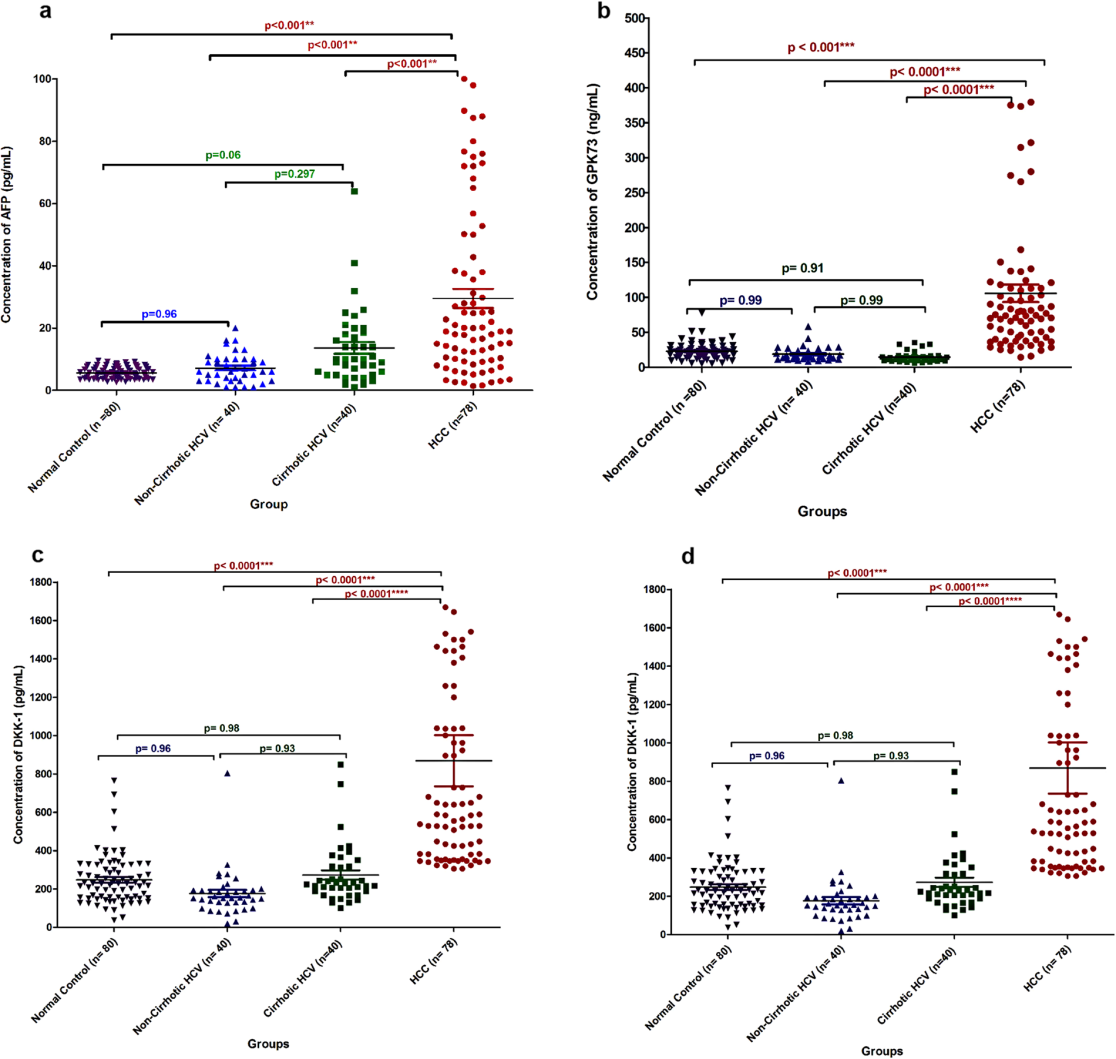
Scatter plots for (**a**) *DDK-1*, (**b**) *GPK73*, and (**c**) *MDK* Levels are presented as ng/ml in hepatocellular carcinoma, cirrhotic, chronic hepatitis and healthy controls.

**Figure 2:**
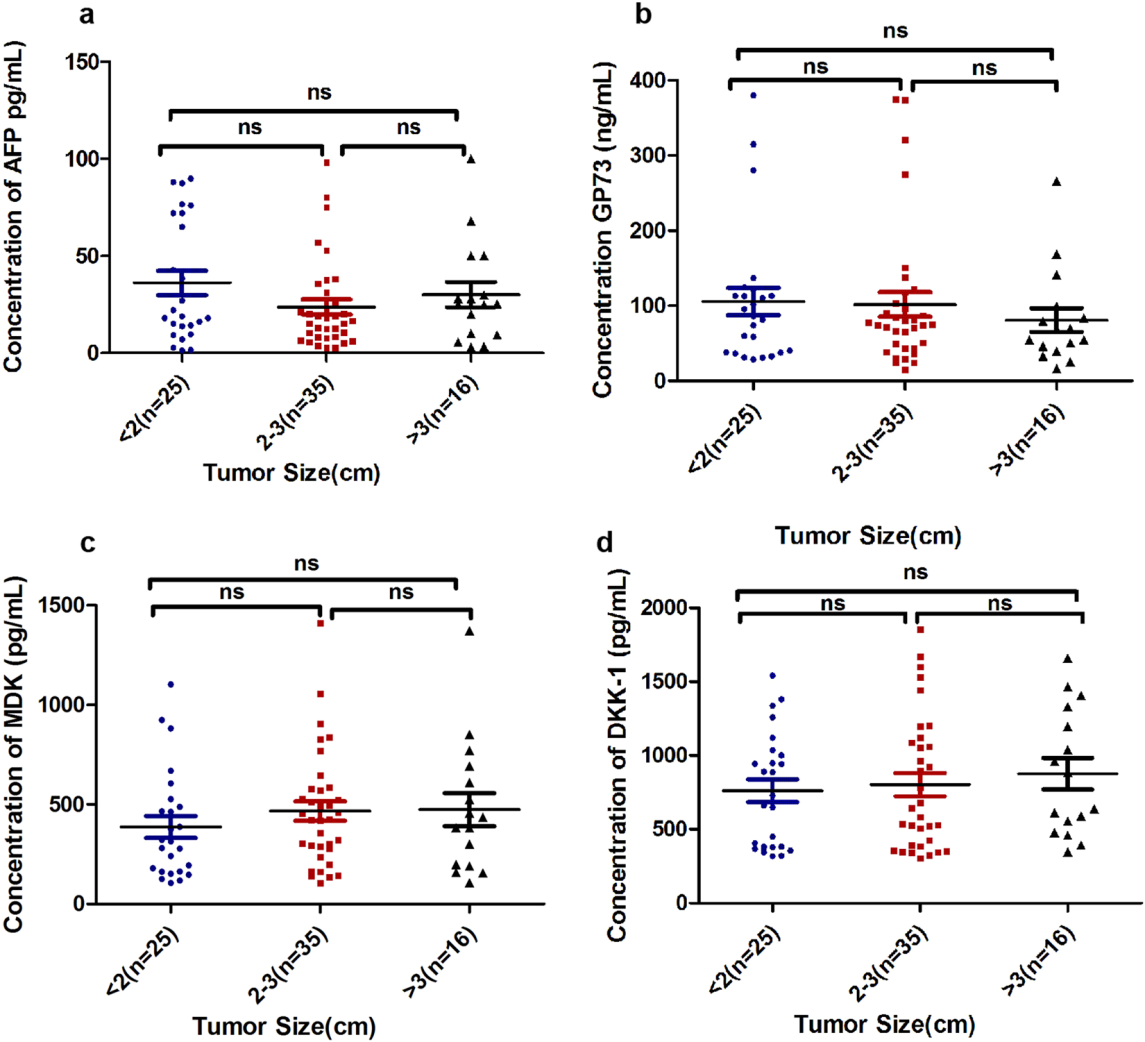
The correlation between serum levels and tumor size in HCC patients.

Serum levels of AFP, MDK, DKK-1, and GP73 were significantly higher in HCC compared to other studied groups (Table 2 and Fig. 1). However; there was no significant difference in serum concentration of the studied biomarkers among cirrhotic HCV patients, non-cirrhotic HCV patients and the healthy control group. Furthermore, the correlation between serum levels and tumor size in HCC patients was assessed as shown in Table 3 and Fig. 2. Serum levels of the four studied markers did not associate significantly with the size of the tumor.

**Table 2:** Serum biomarker levels in all studied groups.

	Mean	Median	Range	IQR	95%CI	Subgroup P* value	
**AFP**							
HCC(n = 78)	29.5 ± 27.1	18.9	1.4–100.0	29.9	23.4–35.6	HCC vs C-HCV	<0.001
C-HCV(n = 40)	13.6 ± 11.99	10.0	1.0–64.0	13.1	9.8–17.5	HCC vs NC-HCV	<0.001
NC-HCV(n = 40)	7.2 ± 4.7	6.0	1.0–20.0	7.0	5.7–8.7	HCC vs CON	<0.001
CON(n = 80)	5.6 ± 1.9	5.5	2.6–9.6	3.4	5.2–6	C-HCV vs NC-HCV	ns
						C-HCV vs CON	ns
						NC- HCV vs CON	ns
**GP73**							
HCC(n = 78)	105.97 ± 112.1	74.2	14.5–741.7	70.9	80.7–131.2	HCC vs C-HCV	<0.001
C-HCV(n = 40)	14.66 ± 7.45	12.6	6.2–35.6	6.4	12.2–17.0	HCC vs NC-HCV	<0.001
NC-HCV(n = 40)	18.9 ± 9.6	15.6	8.5–58.5	10.8	15.8–22.0	HCC vs CON	<0.001
CON(n = 80)	22.88 ± 11.3	21.7	5.53–77.7	9.7	20.4–25.4	C-HCV vs NC-HCV	ns
						C-HCV vs CON	ns
						NC- HCV vs CON	ns
**MDK2**							
HCC(n = 78)	440.0 ± 287.8	382.4	103.77–1410.7	375.4	375.1–504.9	HCC vs C-HCV	<0.001
C-HCV(n = 40)	168.72 ± 167.7	109.5	3.28–605.1	155.7	115.1–222.4	HCC vs NC-HCV	<0.001
NC-HCV(n = 40)	59.9 ± 89.5	34.9	0.89–519.2	65.1	31.3–88.6	HCC vs CON	<0.001
CON(n = 80)	102.9 ± 105.5	76.8	0.1–517.1	101.9	79.4–126.4	C-HCV vs NC-HCV	ns
						C-HCV vs CON	ns
						NC- HCV vs CON	ns
**DKK1**							
HCC(n = 78)	968.99 ± 1240.6	671.3	305.9–1044.29	717.4	689.3–1248.7	HCC vs C-HCV	<0.001
C-HCV(n = 40)	272.8 ± 153.2	226.9	101.4–848.5	129.6	223.9–321.9	HCC vs NC-HCV	<0.001
NC-HCV(n = 40)	176.2 ± 121.2	153.7	20.2–803.8	65.0	137.5–215.0	HCC vs Controls	<0.001
Controls(n = 80)	247.3 ± 128.5	227.6	37.3–764.6	172.1	218.7–275.9	C-HCV vs NC-HCV	ns
						C-HCV vs CON	ns
						NC- HCV vs CON	ns

*Mann-Whitney U test.

C-HCV: Cirrhotic hepatitis C, NC-HCV: non-cirrhotic hepatitis C., IQR: Interquartile range, CI: Confidence Interval, AFP: α fetoprotein, GP73: Golgi Protein 73, MDK: Midkine, DKKpf-1: Dickkopf-1 protein, ns: non-significant.

**Table 3:** Correlation between serum levels of MDK, DKKpf-1, Gp73 and tumor sizes in patients with AFP inHCC infection.

Marker	Tumor size (cm)				P* value
AFP		<2(n = 25)	2–3(n = 35)	>3(n = 16)	0.21
	Mean±SD	36.1 ± 31.4	23.7 ± 23.1	30.0 ± 26.1	
	Median	18.9	16.6	25.2	
	Range	1.4–89.8	2.5–98.0	3.0–100.0	
	95% CI	23.2–49.1	15.8–31.7	16.1–43.9	
GP73	Mean±SD	105.7 ± 90.8	119.9 ± 142.3	80.9 ± 63.7	0.53
	Median	86.4	75.5	61.7	
	Range	28.5–379.9	14.5–741.7	16.1–265.8	
	95% CI	68.1–143.1	71.0–168.8	47.0–114.9	
MDK	Mean±SD	386.9 ± 272.6	466.9 ± 288.1	474.0 ± 331.3	0.52
	Median	314.3	426.2	409.6	
	Range	105.8–1102.1	103.8–1410.7	106.2–1371.8	
	95% CI	274.4–499.4	367.9–565.9	297.5–650.5	
DKK-1	Mean±SD	761 ± 383	1187.1 ± 1789.9	875.4 ± 427.2	0.41
	Median	729.2	680.5	762.2	
	Range	317.8–1541.5	305.9–110442.1	346.4–1658.1	
	95% CI	602.7–919.4	572.4–1802.0	647.7–1103.0	

*ANOVA for association of serum markers levels with tumor size.

AFP: α fetoprotein, GP73: Golgi Protein 73, MDK: Midkine, DKKpf-1: Dickkopf-1 protein.

### Comparison between AUC, sensitivity, and specificity of the biomarkers for the diagnosis of HCC at optimal diagnostic cutoff values

**Figure 3:**
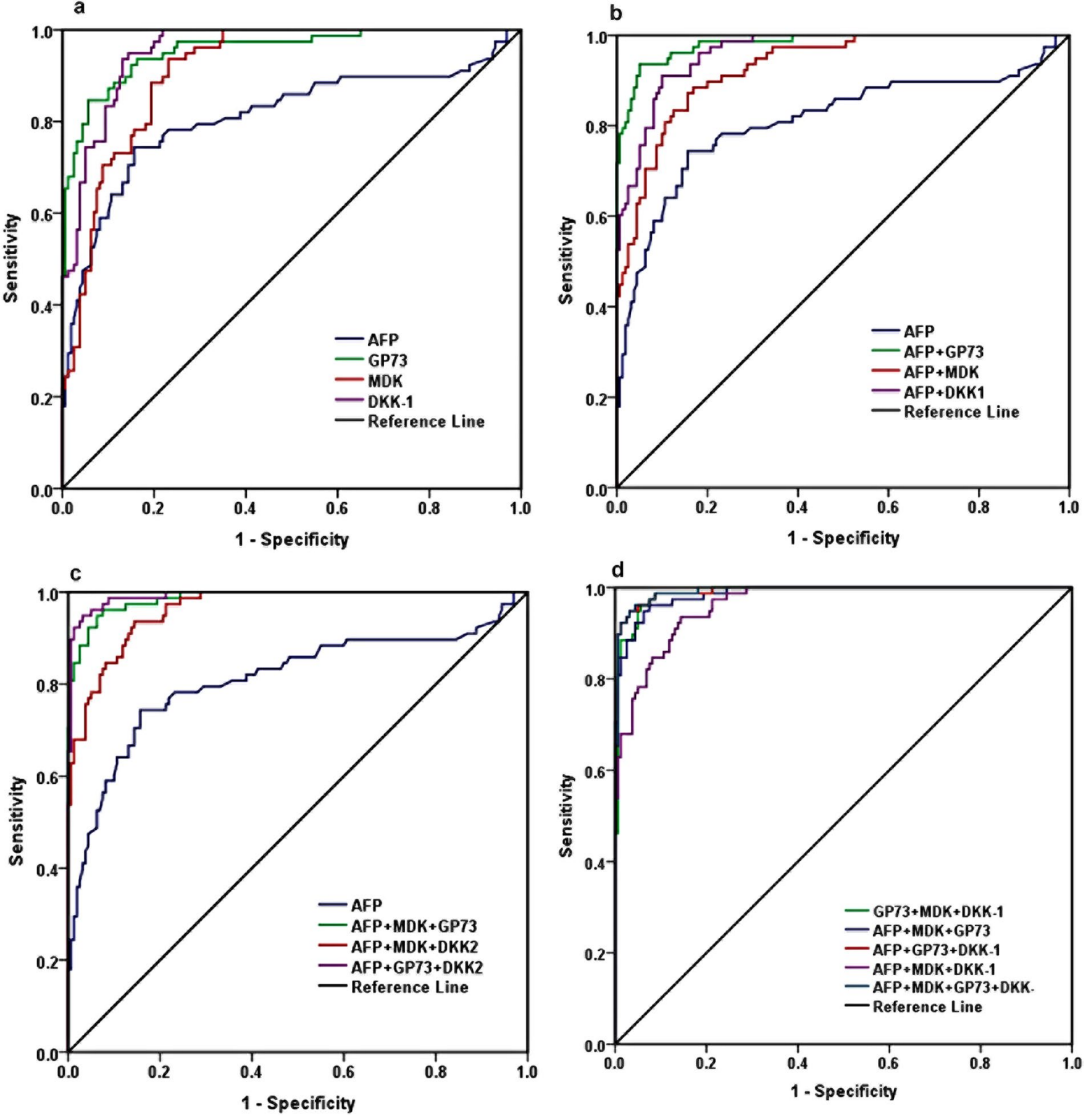
The optimal diagnostic cut off values of AFP, MDK, DKK-1, and GP73 were determined using ROC curve analysis.

**Figure 4:**
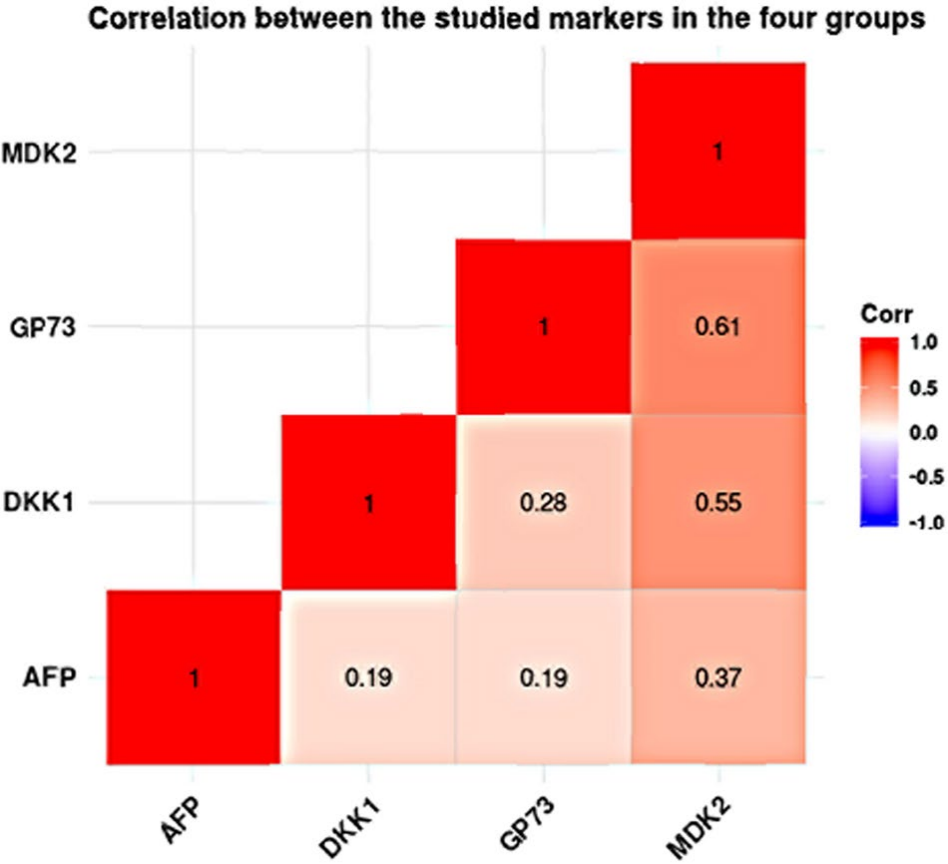
Correlation between the studies markers in the four groups.

The optimal diagnostic cut off values of AFP, MDK, DKK-1, and GP73 were determined using ROC curve analysis (Fig. 3). The cutoff value of AFP was 10.05 ng/mL with 0.81 AUC (95% CI 0.74–0.88), 0.035SE, 74.4% sensitivity and 84.4%specificity. The optimal cut off for GK73 was 29.16 ng/mL with 0.956 (95% CI 0.93–0.98) AUC, 0.014 SE, 91% sensitivity and 85% specificity (P < 0.001). The optimal cut off for MDK, was 152.07 pg/mL with an AUC of 0.91 (95% CI 0.88–0.95), SE of 0.019, a sensitivity of 88.5% and a specificity of 80.6% (P < 0.001). The cut off value of DKK1 was 344.8 pg/mL with an AUC of 0.956 (95% CI 0.93–0.98), SE of 0.011, a sensitivity of 93.6% and a specificity of 86.9% (P < 0.001). The predictive values, accuracy and likelihood ratios of all studied biomarkers for the diagnosis of HCC were calculated according to the cut off values. The diagnostic accuracy of DKK1 (89.08%) was the highest, followed by GP73 (87%) then MDK (83.2%). All three studied biomarkers had a diagnostic accuracy higher than AFP (81%) (Figs. 3 and 4 & Table 4).

**Table 4:** Diagnostic performance of AFP, MDK, DKK-1, and GP73 and their combinations for the diagnosis of HCC patients.

	Sensitivity (%)	Specificity (%)	PPV (%)	NPV (%)	Accuracy (%)	AUC	95%CI	+LR	−LR
***Single Marker***									
AFP	74.4	84.4	69.9	87.1	81.1	0.81	0.74–0.88	4.77	0.303
GP73	91	85	74.7	95	87	0.956	0.93–0.98	6.10	0.110
MDK	88.5	80.6	69	93.5	83.2	0.91	0.88–0.95	4.60	0.14
DKK-1	93.6	86.9	77.7	96.5	89.08	0.956	0.93–0.98	7.15	0.07
***Double Markers***									
MDK + GP73	96.2	87.5	80.6	97.9	91.18	0.975	0.96–0.99	7.69	0.04
MDK + DKK-1	91.0	85.0	79.3	94.0	88.7	0.956	0.93–0.98	6.07	0.11
GP73 + DKK-1	97.4	93.1	87.36	98.7	94.5	0.987	0.98–0.99	14.1	0.027
AFP + MDK	91.0	76.9	66.7	95.38	82.35	0.93	0.90–0.96	3.94	0.12
AFP + DKK-1	91.0	90.0	82.56	95.39	90.76	0.963	0.94–0.98	9.1	0.1
AFP + GP73	96.2	88.1	80.6	97.9	91.18	0.982	0.97–0.99	8.08	0.043
***Triple Markers***									
AFP + MDK + GP73	96.2	92.5	86.2	98.01	93.7	0.987	0.98–1.0	12.8	0.04
AFP + MDK + DKK-1	93.6	85.6	76.04	96.48	88.2	0.964	0.94–0.98	6.7	0.07
AFP + GP73 + DKK-1	98.7	91.2	86.5	99.3	94.5	0.99	0.98–1.0	11.2	0.014
MDK + DKK-1 + GP73	98.7	91.2	86.5	91.2	94.5	0.99	0.98–1.0	11.2	0.014
***Quadruple Markers***									
AFP + GP73 + MDK + DKK-1	98.7	91.2	84.6	99.3	93.7	0.99	0.99–1.0	11.2	0.014

PPV positive predictive value NPV negative predictive value AUC area under the curve LR likelihood ratio AFP: α fetoprotein, GP73: Golgi Protein 73, MDK: Midkine, DKK-1: Dickkopf-1 protein.

### The combination of studied biomarkers for the diagnosis of HCC

A binary logistic regression model was applied to assess the combinatorial ROC curves and to evaluate the diagnostic accuracy of the combinations of AFP, GP73, MDK and DKK3. The new variable predicted probability was created according to the equation obtained by binary logistic regression (HCC versus cirrhotic, non- cirrhotic and healthy controls. The model used in this study was as follows: for the combination of AFP and GP73, Log [p/(1 − p)] = −6.79 + (0.12 × AFP) + (0.125 × GP73), for the combination of AFP and MDK, Log [p/(1 − p)] = −3.61 + (0.076 × AFP) + (0.008 × MDK), for the combination of AFP and DKK-1, Log [p/(1 − p)] = −5.03 + (0.066 × AFP) + (0.008 × DKK-1), for the combination of MDK and GP73, Log [p/(1 − p)] = −5.69 + (0.103 × GP73) + (0.005 × MDK) for the combination of MDK and DKK-1 Log [p/(1 − p)] = −4.88 + (0.005 × MDK) + (0.008 × DKK-1), for the combination of GP73 and DKK-1, Log [p/(1 − p)] = −7.39 + (0.099 × GP73) + (0.007 × DKK-1), for the combination of AFP, GP73 and MDK, Log [p/(1 − p)] = −7.21 + (0.105 × AFP) + (0.113 × GP73)+(0.004 × MDK),), for the combination of AFP, MDK and DKK-1, Log [p/(1 − p)] = −5.49 + (0.065 × AFP) + (0.005 × MDK) + (0.007 × DKK-1), for the combination of AFP, GP73 and DKK-1, Log [p/(1 − p)] = −8.6 + (0.097 × AFP) + (0.106 × GP73) + (0.006 × DKK-1), for the combination of GP73, MDK and DKK-1, Log [p/(1 − p)] = −7.5 + (0.095 × GP73) + (0.002 × MDK) + (0.007 × DKK-1) and for the combination of all four markers,Log [p/(1 − p)] = −8.62 + (0.096 × AFP) + (0.105 × GP73) + (0.001 × MDK) + (0.006 × DKK-1).

The new variable was used for ROC curve analysis in order to assess whether the combined use of AFP, GP73, MDK and DKK-1 was better than the use of any of these biomarkers alone. The AUC value for the combination of GP73 and DKK-1 was the highest (0.987, 95% CI 0.98–0.99) with 97.4% sensitivity and 93.1% specificity followed by AUC value for the combination of GP73 with AFP (0.982, 95% CI 0.97–0.99, 96.2% sensitivity, 88.1% specificity) or MDK (0.975, 95% CI 0.96–0.99, 96.2% sensitivity, 87.2% specificity).

For triple markers combinations, the AUC was the largest for the combination of DKK-1 with GP73 and AFP(0.99, 95% CI 0.99–1.0, 98.7% sensitivity, 91.2% specificity) or with GP73 and MDK(0.99, 95% CI 0.99–1.0, 98.7% sensitivity, 91.2% specificity). A similar result was shown for the combination of the four markers (AUC 0.99, 95% CI 0.99–1.0, 98.7% sensitivity, 91.2% specificity) (Fig. 3, Table 4). These findings suggest that the three markers panel (GP73, MDK and DKK-1) with or without AFP could improve the diagnostic efficacy for discriminating HCC patients from cirrhotic and non-cirrhotic hepatitis C virus carriers.

### Correlation of studied markers in all studied groups and in HCC

**Figure 5:**
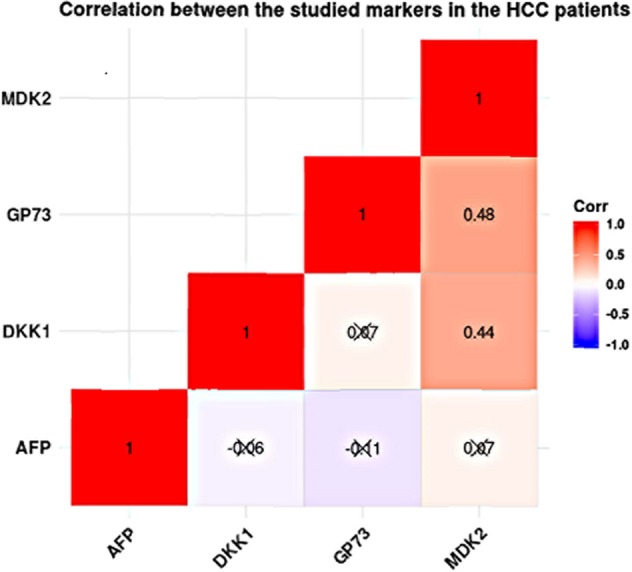
Correlation between the studies markers in the HCC patients.

In all studied groups, there was strong positive correlation between MDK and GP73 (r = 0.612; p.<0.001). In addition, DKK-1 was positively correlated to GP73 (r = 0.282, p < 0.001) and MDK (r = 0.547, p < 0.001). In HCC group, serum MDK levels were significantly correlated to GP73 (r = 0.441, p < 0.001) and to DKK-1 (r = 0.482, p < 0.001). However, no significant correlation was detected between GP73 and DKK1 (r = 0.066, p = 0.565) (Fig. 5 & Table 5).

**Table 5:** Correlation analysis of studied markers in all studied groups and in HCC patients.

	MDK	DKK-1	GP73
**Overall population (n = 238)**			
MDK	—	r = 0.547 P < 0.001	r = 0.612 P < 0.001
DKK-1	r = 0.547 P < 0.001	—	r = 0.282 P < 0.001
GP73	r = 0.612 P < 0.001	r = 0.282 P < 0.001	—
**HCC (n = 78)**			
MDK	—	r = 0.441 P < 0.001	r = 0.482 P < 0.001
DKK-1	r = 0.441 P < 0.001	—	r = 0.066 P = 0.565
GP73	r = 0.482 P < 0.001	r = 0.066 P = 0.565	—

r = rho Pearson correlation coefficient, GP73: Golgi Protein 73, MDK: Midkine, DKK-1: Dickkopf-1 protein

rho value range is (0.2–0.5) between MDK, andGP73(r = 0.612; p < 0.001 & rho value > 0.5) (Supplement-1).

In the HCC group, there was a moderately positive correlation between MDK& DDK-1(r = 0.0441) as well as between MDK and GP73(r = 0.482; p < 0.001). On the other hand, either a weakly positive correlation or no correlation was detected between MDK, and GP73 [r = 0.066; p = 0.565; rho value <0.2; (Supplement-1) No significant correlation was found between GP73& DKK-1(r = 0.066; p = 0.565).

On the other hand, our data did not showed any significant correlation between tumor size and all tested markers contrasts with some previous data in literature which showed that DKK-1 protein level correlates significantly with tumor size especially in HCV and/or HBV infected(Supplement-2).

## Discussion

In the last few years many proteins have been addressed as potential biomarkers for diagnosing HCC that replaced AFP in order to increase its diagnostic accuracy, either alone or combined with it. Some of these markers are increasingly used in the routine workup of the patients such as Lectin-bound alpha-fetoprotein (AFP-L3) and DCP (des-gamma-carboxyprothrombin) which is also known as prothrombin induced by Vitamin K Absence II (PIVKA II). At the same time, there are several other new markers which are still under research including Dickkopf-1 (DKK1), Golgi Protein-73 (GP73), Glypican-3(GPC3), and different microRNAs (miRs) such as miR-29a, miR-29c, miR-133a, miR-143, miR-145, miR-192, and miR-505)^7^. The aim of the current study was to evaluate the possible role(s) of serum levels of GP73, MDK, DKK-1 proteins for early detection of HCC in chronic HCV infected patients, and to compare their diagnostic accuracy either with AFP or alone.

In the current study MDK levels were significantly higher in HCC group compared to the other studied groups. However there was no significant difference in the MDK protein expression level between the other two groups. This indicates that the diagnostic accuracy of MDK for HCC diagnosis is high and that it can also differentiate between HCC and non-HCC cases effectively irrespective of the presence of cirrhosis. This also shows that MDK may not be related to the inflammatory process and accordingly, it could be considered as an excellent, non- invasive diagnostic marker for HCC rather than the AFP. Our findings are in agreement with recently published reports from Europe and USA^13,16,17^. However, these data differs from other reports in literature which recommended the combination of MDK with AFP in HCC detection^17^. Our results also show that this combination of MDK with AFP enhances the sensitivity, specificity and accuracy, AUC and the 95% CI.

DKK-1 is another biomarker which was significantly higher in the HCC patients compared to other groups. However, there was no statistically significant difference in its levels across the non-HCC groups. This also confirms the important diagnostic role of DKK-1 in HCC patients and consequently it could be utilized in distinguishing HCC from other premalignant lesions as well as for different stages of chronic liver disease. This is in agreement with the study of Shen *et al*.^18^, and Geet *et al*.^19^, however they contrast with the previously reported data of Jang *et al*., 2016 who mentioned that AFP is still considered the most valuable and accurate marker for diagnosing HCC. In the contrary, our results contrast with those of Jang ES., 2011^20^ who showed that a combination of DKK-1 and AFP increases the sensitivity of DKK-1 but it reduces it’s the specificity, accuracy, AUC, 95% CI.

The GP73 is expressed in the biliary epithelial cells and hepatocytes and its increase is always related to the degree of liver injury. It has also been shown that, the amount of GP73 differs according to the degree of fibrosis or inflammation in the studied subjects^21^. We found that, serum GP73 was significantly elevated in HCC group compared to other groups though it was not significantly higher in the cirrhotic group compared to the non-cirrhotic group. This could be explained, at least partially by the fact that the amount of GP73 secreted by the damaged hepatocytes and the activated stellate cells depend on the degree of cell injury and fibrosis. Thus, lower concentrations of GP73 in cirrhotic compared to the non-cirrhotic group might be related, at least partially, to the inability of the cirrhotic liver to produce proteins including the GP73 protein. This could support the important role of GP73 as a non-invasive serum biomarker for HCC diagnosis which high diagnostic accuracy. According to our data GP73showed better sensitivity and specificity for predicting HCC compared to AFP. Our data in this context support the previously published data of Mao Y. *et al*.^22^, Li B., *et al*.^23^, and Chen, 2012^18^ who provided evidence that GP73 can act in combination with AFP in order to increase its specificity and sensitivity as well as its diagnostic accuracy. Taken together, this could highlight the benefit of using a combination of AFP and GP73 in order to improve the diagnostic accuracy compared to either of them alone. However, these findings contrasts with those of Özkan H *et al*.^24^, who found that GP73 has specificity and sensitivity lower than AFP as well as with Qiao Y. *et al*., 2014 and Liu T.^25,26^, who reported that GP73 is not a suitable marker for the detection of HCC because its elevation is not only related to HCC patients but also to cirrhotic patients.

According to our data a triple markers panel formed of GP73, DKK-1 and MDK have 100% sensitivity and 65.6% specificity, however the addition of AFP to this panel decreases the specificity to 54.4% with no added value to the sensitivity. The presence of Gp73 provides the best sensitivity and specificity in different double and/or triple combinations. We were not able to compare our markers’ combinations with other studies since (1) we did not find previous studies in literature using the same markers panel used by our group and (2) there was no study in literature that compared between these three markers, and AFP, for the early detection of HCC (Supplement-3).

By comparing our four chosen markers (AFP, GP73,MDK and DKK-1), in terms of sensitivity, specificity, AUC, and accuracy, we found thatDKK-1 protein is the best single studied biomarker for detecting (diagnosing) HCC, which is in agreement with previous studies^18–20^. However, by making different markers’ combinations (double, triple, and quadruple), we found that the sensitivity increases to approximately 100% with compromising the specificity which reaches approximately 50%. Accordingly, we concluded that, the best double markers panel to be used includesGP73 and DKK-1 which provides 100% sensitivity, 74.4% specificity as well as AFP + GP73 which has 98.7% sensitivity and 76.2% specificity.

## Conclusion

We conclude that serum levels of MDK, DKK-1, Gp73 were significantly higher in HCC patients compared to the non-HCC group. However, the best prediction value is provided by the combination of DKK-1 with Gp73as it showed high accuracy (82.8%) and specificity (74.4%). However, this preliminary study should be confirmed in a larger study including more patients from each studied group.

## Supplementary information

Supplementary Dataset 1.

Supplementary Dataset 2.

Supplementary information.
