# Supplementary material for: The possible role of Dickkopf-1, Golgi protein- 73 and Midkine as predictors of hepatocarcinogenesis: a review and an Egyptian study
Source: Sci Rep. 2020 Mar 20;10:5156. doi: 10.1038/s41598-020-62051-6 (PMC7083902; doi:10.1038/s41598-020-62051-6)
Supplement: Supplementary file 3 — Supplementary information. [file 41598_2020_62051_MOESM3_ESM.docx]

**Supplementary 3: literature that compared between these three markers for the early detection of HCC**

| **The possible role of Dickkopf-1, Golgi protein- 73 and Midkine as**  **predictors of hepatocarcinogenesis: a review and an Egyptian study**    Abdel-RahmanNabawyZekri^1^,Mohamed El Kassas^2^, El Sayed TarekAbd El Salam ^3^, Reem Mostafa Hassan ^4^, MarwaMohanad^5^, Reham Mohamed Gabr^1^, Mai M. Lotfy^1^, Rania A. Talaat Abdel-zaher^3^.Abeer A. Bahnassy^6^,Ola S.Ahmed^1^. | | | | | | |
| --- | --- | --- | --- | --- | --- | --- |
| **Type of marker** | **Investigator** | **Country** | **Sample Type** | **Studied groups** | **Technique Used for Detection** | **Results** |
| **Dickkopf-1 (DKK-1)** | (Watany *et al*, 2017) | Egypt | Serum | 1-HCC  2- HCC with positive HCV antibodies  3- HCC with positive HCV antibodies and positive anti-schistosomal antibodies  4-Healthy | 1-Enzyme-linked immunosorbent assay (ELISA) of serum MDK  2- Reverse Transcriptase– Polymerase Chain Reaction of Midkine gene expression for HCC and healthy groups | 1-DKK-1 expression gene in HCC with previous schistosomal infections group was higher significance than other groups (p<0.001). |
|  | (Qin *et al*, 2017) | China | Serum | 1-HCC  2- Non-HCC  3- Control | Enzyme-linked immunosorbent assay (ELISA)  of serum MDK | 1- DKK-1 concentration were significantly higher in HCC group than other groups (<0.05), and slightly higher but not statistically different in non-HCC than normal group.  2- DKK-1 was more accurate in diagnosis HCC where its specificity, sensitivity 44%, 72.3% respectively than AFP which its specificity, sensitivity 58.1%, 29% respectively where that differences were significant (p=0.04).  3- DKK-1combination with DCP, and AFP achieved higher specificity and sensitivity 78%%, and 93.02% than using each one alone where that elevation was statistically different (p<0.05). |
|  | (Fouad etal, 2016) | Egypt | Serum | 1-HCC  2- Liver cirrhosis  3- Chronic HCV | Enzyme-linked immunosorbent assay (ELISA)  of serum MDK | 1- DKK-1 concentration were significantly higher in HCC group than liver cirrhosis group (<0.05), but there was no significant difference with chronic HCV group (<0.3).  2-DKK1 concentration was correlated with number of focal lesions, portal vein thrombosis, and tumor size in advanced hepatocellular carcinoma (diameter>5 cm), but not correlated with tumor size smaller than 3 cm in diameter.  3- DKK-1was more accurate in diagnosis HCC where its specificity, sensitivity 89.3%, 67.5% respectively than AFP which its specificity, sensitivity 76.7%, 65.4% respectively. |
|  | (Jang et al., 2016) | France | Serum | 1-HCC  2- Liver cirrhosis | Enzyme-linked immunosorbent assay (ELISA)  of serum MDK | 1-DKK-1 concentration was highly significant different in HCC group than liver cirrhosis group p (<0.001).  2-AFP had the best biomarker than other biomarkers PIVKA-II, OPN, and DKK-1for HCC diagnosis.  3- Combination of AFP with DKK-1 showed an increased Sn (78.4%), but a decreased Sp (72.5%) as compared to AFP alone while AFP with PIVKA-II showed (Sp 87.6%, Sn 63.5%), representing the highest Sn and Sp among all the 2-marker combinations.  4- Combination of 4 biomarkers (AFP, OPN, DKK-1 and PIVKA-II) had Sn (85.1%) and Sp (54.9%) was not improved.  5- DKK-1 seemed to be a promising complementary marker in conjunction with AFP, especially in early stage HCC patients whose AFP level is<20 ng/mL. |
|  | (Kim et al., 2015) | Korea | Serum | 1-HCC  2- Liver cirrhosis  3- chronic HCV  4-Healthy | 1-Transcription polymerase chain reaction (RT-PCR)  2- Wound healing assays  3- Invasion assays  4- Enzyme-linked immunosorbent assay (ELISA)  of serum MDK | 1-DKK-1 concentrations were significantly higher in all HCC patients (patients who underwent surgical resection) and those (who were treated with other modalities) than in the three control groups [healthy subjects, patients with chronic hepatitis, and those with liver cirrhosis p< 0.001.  2- AFP and DCP concentrations.  Were similar to the DKK-1 results where both were significantly higher in HCC patients than in the control groups.  3- DKK-1 level in early-stage HCC patients (graded TNM I–II and BCLC A–B) were significantly higher than control p<0.001  4- AFP and DCP levels were significantly different between controls and TNM I–II (p=0.038 and p<0.001) and BCLC A–B stage patients (p=0.046 and p<0.001).  5- DKK-1 had a greater ability for identifying HCC from the controls than AFP and DCP where AUC (0.829 vs. 0.794 and 0.815, respectively).  6- The combination of DKK-1with AFP or DCP was significantly higher than DKK-1 alone.  7-The triple combination (DKK-1 plus AFP plus DCP) showed the best accuracy (AUC=0.939 for TNM and 0.940 for BCLC, respectively). |
|  | (Ge et al., 2015) | China | Serum | 1-Early HCC  2- HCC  3-Liver cirrhosis  4- Chronic hepatitis B  5- Healthy control | Enzyme-linked immunosorbent assay (ELISA)  of serum MDK | 1- DKK1 concentration, AFP, and OPN were significantly elevated in patients with HCC when compared to all three control groups. while AFP, OPN were significant higher in other 3groups but DKK-1 was not.  2- DKK-1was more accurate in diagnosis HCC where its specificity, sensitivity 89.37 %, 79.78 % respectively than AFP which its specificity, sensitivity 88.04 %, 71.91 % respectively, and also more accurate than OPN which its specificity, sensitivity were 82.06%, 89.89 respectively.  3- The combination of the three markers showed the largest AUC when compared with any single marker alone which mean that their combination could improve the diagnostic value in distinguishing patients with HCC from all groups including health individuals, LC patients, and chronic hepatitis B virus carriers.  4- DKK1 was better in distinguishing HCC, especially patients at an early stage, from high-risk patients  5- The combination of three markers had no significantly different AUC, sensitivity, and specificity values when compared with the combination of AFP and DKK1 in differentiating patients with early-stage HCC from high- risk controls.  6-The correlation between 3 markers showed that Serum AFP level was found to be significantly associated with cirrhosis, and serum OPN level was found to be significantly associated with HBsAg copies, while DKK1 had no statistically significant correlation with all these pathology parameters.  7-DKK-1concentration was correlated with number of focal lesions, and tumor size |
|  | (Shen et al., 2012) | China | Serum | 1-Early HCC  2- HCC  3-Liver cirrhosis  4- Chronic hepatitis B  5- Healthy control | Enzyme-linked immunosorbentassay (ELISA)  of serum MDK | 1-DKK1 concentration was significantly higher in patients with HCC than in all other controls.  2- DKK1 had diagnostic value for HCC better than that of AFP, especially for patients with AFP-negative status and early-stage of HCC.  3- DKK-1 combination with AFP improved its diagnostic ability for HCC infection.  4- DKK1concentrations in serum correlated with tumor size where  (p=0·001), butnot with BCLC stage  5- DKK1improved differential diagnosis of early-stage HCC from  all controls and from controls at risk of HCC, compared with AFP  6-The combination ofDKK1and AFP increased the diagnostic accuracy for HCC compared with either test alone.  7- DKK1 concentrations in serum decreased after surgery that refer to ability for using this protein as  surveillance biomarker to assess the therapeutic response of HCC patients. |
|  | (Tung et al., 2011) | China | Serum | 1-Advanced HCC  2-Early HCC  3-HCC after treatment  4-Cirrhosis  5- HBV carriers | 1-Real-time quantitative PCR (qPCR) assay  2- Gene expression microarray assay  3- Enzyme-linked immunosorbent assay (ELISA)  of serum MDK | 1- DKK1 transcript in human HCCs was significantly upregulated when compared with the corresponding non-tumorous livers (P < 0.001).  2- DKK1 concentration was significantly higher in the two HCC groups, when compared with the HBV carrier and cirrhosis groups (P < 0.001), while in the HBV carrier group it was not significantly different from that of the cirrhosis group.  4- DKK1 concentration was also significantly higher in the advanced HCC group than the early HCC one (P < 0.012), and significantly lower in the group of HCC patients after treatment when compared with advanced HCC group (P < 0.001).  5- DKK1concentration was correlated with tumor size where  (p=0·001), but not with BCLC stage except it’s highest (p=0·07). |

|  |
| --- |

| **Type of marker** | **Investigator** | **Country** | **Sample Type** | **Studied groups** | **Technique Used for Detection** | **Results** |
| --- | --- | --- | --- | --- | --- | --- |
| Golgi protein 73 (GP73) | (Li et al.,2017) | China | Serum | 1-HCC group  2- Non HCC group | Enzyme-linked immunosorbent assay (ELISA) of serum GP73 | 1-Gp73 has a certain guiding significance to  Predict the risk of HCC in hepatic cirrhosis patients where its concentration decreased gradually with advanced HCC (p=0.041). In non HCC group was 194.6 (66.12–350) ng/ml in hepatic cirrhosis (non-HCC), and 154.2 (13.14–275.4) ng/mL in HCC.  2-AFP, AFP-L3, and ALT had good  predictive power for HCC detection; AUC were 0.736, 0.744, and 0.693, respectively  3- The best combination was ALT and AFP.  4- The combination of three markers AFP, AFP-L3, and ALT could not significantly improve the prediction efficacy of HCC. |
|  | (Mabood et al., 2017) | Egypt | Serum | 1-HCC  2- Cirrhosis | Enzyme-linked immunosorbent assay (ELISA) of serum GP73 | 1-GP73 was highly significant different in HCC individuals than Cirrhosis group.  2-There was no correlation between concentration of Gp73 and size of focal lesions.  3- The diagnostic accuracy of Gp73 was improved by its combination with AFP. |
|  | (T. Liu et al., 2017) | China | Serum | 1-HCC  2-pre-cirrhosis  3- Cirrhosis | Enzyme-linked immunosorbent assay (ELISA) of serum GP73 | 1-Gp37 in HCC individuals was highly significance than other groups  2- Gp73 concentration in cirrhotic patients was higher than that in non-cirrhotic  3- GP73 elevation in HCC individuals may be related to cirrhotic background so it failed to distinguish HCC patients.  4- There was no correlation between the serum levels of GP73, and tumor size or tumor node metastases (TNM) or degree of tumor differentiation. |
|  | (Y. Zhang et al., 2016) | China | Serum | 1-liver Cancer  2- Liver disease  3- Normal | 1- Indirect Enzyme-linked immunosorbent assay (ELISA) of serum GP73  2- Western blot assay | 1-GP73 is an excellent marker for diagnose healthy from liver cancer patients where its specificity, and sensitivity in liver cancer patients were 97%, and 87% respectively but 76%, 97% respectively in liver disease patients.  2- Gp73 concentration were significance differentiated in both groups Liver cancer, and liver disease than in normal group (P<0.0001).  3- the activity of Gp73 alone is limited so its combination with other biomarker as AFP is desirable  4- Developed antibodies can be used as ideal reagents for detection of biomarkers. |
|  | (Z. Zhang, Zhang, Wang, Xu, & Xu, 2015) | China | Serum | 1-HCC group  2- Non-HCC group  3- Healthy group | Enzyme-linked immunosorbent assay (ELISA) of serum GP73 | 1-Gp73 concentration was significantly higher in HCC group than non-HCC, and healthy group while Gp73 had not any significant difference between non-HCC group and healthy one  2- GP73 is better than AFP-L3 where its sensitivity, specificity, and accuracy were 66.0%, 96.2%, and 84.6%, respectively; while in AFP-L3 were the sensitivity, specificity, and accuracy of AFP-L3 were 50.0%, 97.5%, and 79.2%, respectively.  3- Its combination with AFP-L3 achieved higher specificity and accuracy100.0%, and 76.9% than using each one alone but the sensitivity decreased 40.0%. |
|  | (Wang et al., 2014) | China | Serum | 1-HCC  2- Cirrhosis  3-HBV carriers  4- Healthy | Immunobloting  assay | 1-GP73 concentration was significantly higher in HCC patients than that in other non-HCC groups (P < 0.05).  2- GP73 had great advantage in differentiating HCC from cirrhosis patients.  3- GP73 showed a steady elevated level throughout all the sizes. This result suggests that GP73 is a better marker than AFP for diagnosis of small HCC from cirrhosis.  4- Its combination with AFP significantly increases the diagnostic sensitivity and specificity of HCC. |
|  | (Jia et al., 2014) | China | Serum | 1-HCC  2- Benign liver diseases  3- Healthy | Enzyme-linked immunosorbent assay (ELISA) of serum GP73 | 1-Gp73 was distinguished than AFP, and AFP-L3 by its ability for detection of HCC at low cutoff value <20 ng/ml.  2- Its combination with AFP-L3 increases  the sensitivity and specificity in diagnosis of HCC especially in patients with AFP<400 ng/ml. |
|  | (Chen, 2013) | China | Serum | 1-HCC  2- Liver Cirrhosis  3-Chronic hepatitis  4- Healthy group | - Enzyme-linked immunosorbent assay (ELISA) of serum GP73 - Time-resolved fluorescence immunological assay (TRFIA) of serum GP73 | 1-Serum Gp73 in HCC individuals were higher than chronic hepatitis, liver cirrhosis, and healthy individuals.  2-There was no correlation between Gp73 concentration, and tumor size or grading.  3- The diagnostic accuracy of Gp73 was improved by its combination with AFP  4- The sensitivity of ELISA technique for GP73 is lower than that of TRIFA |
|  | (Zhou et al., 2012) | China | Serum | 1-HCC  2- Chronic liver diseases (CLD)  3- Liver cirrhosis (LC) | Enzyme-linked immunosorbent assay (ELISA) of serum GP73 | 1-GP73 concentration were higher in HCC than AFP  2- Gp73 accuracy in HCC detection was better than AFP where sensitivity and specificity (77.1%, 82.6%) for GP73, (28.8%. 59.8%) forAFP  3- Combination of GP73 and AFP had much higher but not significantly differences of the sensitivity and specificity than using GP73 alone for HCC diagnosing. |
|  | (Mona A El Shafie1 etal.,  2012) | Egypt | Serum | 1-HCC  2- Cirrhosis  3- Control | Enzyme-linked immunosorbent assay (ELISA) of serum GP73 | 1- GP73 were significantly elevated in LC and HCC patient groups than control group and more elevated in HCC group than in LC group and control group (p<0.001).  2- GP73 was more accurate in diagnosis HCC with specificity, sensitivity 95%, 87% than AFPwith specificity, sensitivity 60%, 77.4%.  3-Combination of GP73 and AFP had higher accuracy than using every one alone wherespecificity, sensitivity were 90%, 90.3% respectively. |
|  | (Tian et al., 2011) | China | Serum | 1-HCC  2-Liver cirrhosis  3-Hepatitis  4-Control | 1- Enzyme-linked immunosorbent assay (ELISA) of serum GP73  2- Western blot analysis of serum Gp73 | 1-Gp73 in all groups were highly significance than control group  2- GP73 was significantly higher in liver cirrhosis group than HCC group that may be related to degree of fibrosis or inflammation of subject  3-There were not significantly difference between early and advanced HCC infection  4- There is correlation between the Gp73 and tumor size  5- Finally Gp73 elevation related to hepatic deterioration, and chronic fibrosis and its combination with AFP help in differentiation between hepatic diseases |
|  | (X. Liu et al., 2011) | China | Serum | 1-HCC  2- Liver fibrosis  3- HBeAg negative CHB  4- HBeAg-positive CHB  5- Healthy | 1- Enzyme-linked immunosorbent assay (ELISA) of serum GP73 | 1- GP73concentration increased  significantly in the HBeAg-positive CHB group (p-0.001), HBeAg-negative CHB group (p-0.001), liver fibrosis group (p-0.001) and HCC group (p-0.001).  2- GP73 concentrations were increased significantly in the HBeAg-positive CHB group than the HBeAg-negative group (p=0.001).  3- GP73 concentrations were significantly increased in the liver fibrosis group than healthy (p-0.001).  4- GP73 value in the HCC group was  Significantly higher than that in the HBeAg positive CHB group (p-0.001), HBeAg-negative CHB group (p-0.001), and liver fibrosis group (p=0.009).  5- There was significantly positive correlation between GP73 and AFP (p=0.037).. |
|  | (Morota et al., 2011) | Japan | Serum | 1-HCC  2- Liver cirrhosis  3- Chronic hepatitis  4- Healthy | Enzyme-linked immunosorbent assay (ELISA) of serum GP73 | 1- GP73 concentration in the HCC group was not significantly different from that in the cirrhosis group (p=0.4121), but its concentration in the cirrhosis group was significantly higher than that seen in the hepatitis group and normal group (p-0.0001).  2- GP73 concentration was significantly higher in the HCV infected cirrhosis group than the HCV -infected group (p0.0001) while there is no significantly different in the cirrhosis group with HBV infection than that with HBV infection (p=0.3239)  3- Gp73 had lower accuracy against HCC than AFP where their sensitivity were 88.6%, 62.9% respectively, and specificity 61.5%, 92.3% respectively. |
|  | (Mao et al., 2010) | China | Serum | 1-HCC  2- Liver cirrhosis  3- Healthy | Immunobloting assay | 1-Serum GP73in HCC individuals was higher than that with cirrhosis , and healthy individuals  2- Serum Gp73 had a higher specificity and sensitivity than AFP in HCC diagnosis  3- There was no correlation between GP73 concentration, and tumor size or Child-pugh class.  4- Gp73 concentration in patients with other types of benign or malignant liver cirrhosis were significantly lower than those with HCC so it is useful in detection the nature of Hepatic tumors.  5- The diagnostic accuracy of Gp73 was improved by its combination with AFP |

|  |
| --- |

| **Type of marker** | **Investigator** | **Country** | **Sample Type** | **Studied groups** | **Technique Used for Detection** | **Results** |
| --- | --- | --- | --- | --- | --- | --- |
| **Midkine**  **(MDK)** | (Hodeib etal, 2017) | Egypt | Serum | 1-HCC on top of cirrhosis  2- HCV without cirrhosis  3- HCV with liver cirrhosis  4- Healthy | Enzyme-linked immunosorbent assay (ELISA) of serum MDK | 1- MDK concentration was significantly elevated in HCC patients than other studied groups (p<0.001)  2- MDK concentration had non-significant correlations with other laboratory parameters in all the studied groups (p>0.05) and AFP.  3- MDK had better sensitivity and specificity than AFP in the diagnosis of HCC, Sn (98.4% vs.97%), Sp (96.2% vs. 95%).  4- MDK combination with AFP yielded a diagnostic value of (98%) in the diagnosis of HCC. |
|  | (Vongsuvanh et al., 2016) | China | Serum | 1-HCC  2- Liver chronic  3- Cirrhotic  4- Healthy | Enzyme-linked immunosorbent assay (ELISA) of serum MDK | **A-Cross sectional study**:  1-MDKwas significantly higher in HCC than other groups (p<0.001).  2- MDKwas associated with tumour characteristics as poor Child-Pugh status (P = 0.01), advanced BCLC stage (P = 0.006),vascular invasion (P = 0.007) and high tumour number (P = 0.007).  3- AFP may play important role as superior for MDK in HCC diagnosis at advanced and early stages.  4-AFP also had superior diagnostic performance compared to the MDK in distinguishing HCV or HBV-associated HCC from cirrhosis.  5- Combination of MDK with AFP did not signifi- cantly improve the diagnosis of HCC compared to either test alone.  6-MDK had ability in detection of HCC in HCC-patients with normal AFP.  7-MDKhad ability to differentiate NASH-HCC from non-malignant liver disease  **B- Longitudinal Study**:  MDK may have a role in the pre-clinical diagnosis of HCC |
|  | (Shaheen etal, 2015) | Egypt | Serum | 1-HCC  2- Liver cirrhosis  3- Healthy | Enzyme-linked immunosorbent assay (ELISA) of serum MDK | 1-MDK concetration was significantly higher in HCC than other groups (p<0.001), and higher in cirrhotic group than healthy but not significance.  2- MDK concentration had no-significant correlation with BCLC stage, tumor diameter and number of tumor nodules.  3- MDK concetration had no- significant correlation with serum levels of AFP in all studied patients.  4- MDK had better sensitivity, specificity, and accuracy than AFP in the diagnosis of HCC Sn(92.5% vs.40%), Sp (83.3% vs 53.3%), and accuracy (88.5% vs 64.2%).  5-MDKconcentration had no significant correlation withBCLC stages, tumor size, number of lesions or serum levels of AFP. |
|  | (Saad et al., 2013) | Egypt | Plasma | 1-HCC  2- Liver cirrhosis  3- Chronic HCV | Quantitative RT-PCR measured in peripheral blood mononuclear cells | 1-MDK gene expressions were significantly higher in the HCC group compared to the liver cirrhosis and CHC group (p-0.022, 0.01, <0.01, <0.01, respectively).  2- MDK gene expresion had no significant correlation with Child Pugh score, number of tumor nodules, the size of the lesions, extra hepatic spread or the AFP level.  3-MDK had higher specificity, and sensivity than AFP. |
|  | (Zhu et al., 2013) | China | Serum | 1-HCC  2- Liver cirrhosis  3- Benign liver tumor  4- Gastrointes- tinal malignant tumor  5- Healthy | Enzyme-linked immunosorbent assay (ELISA) of serum MDK | 1-Elevated expression of MDK was observed in hepatocellular carcinoma cell lines as well as in culture medium compared with that in normal liver cell lines and that elevation was significance (P < 0.001).  2- MDK expression in tumor tissues positively correlated with the serum samples when MDK protein expression levels in hepaotcellular carcinoma tissues paired with serum samples.  3- MDK senstivity, and specicificity were significantly higher than AFP. Moreover, multivariate logistic regression model showed the combination of MDK and AFP could improve the diagnostic performance significantly.  4- MDKconcentration had no significant correlation withBCLC stages,  5- serum MDK had an outstanding performance for distinguishing AFP-negative hepatocellular carcinomas from non–hepatocellular carcinoma controlsincluding liver cirrhosis patients.  6-MDK could be a sensitive tumor  marker to monitor the treatment response and postoperation tumor recurrence in patients with hepatocellular carcinomas |
|  | (Hung et al., 2011) | China | Serum | 1-HCC  2- Non-HCC | 1-Enzyme-linked immunosorbent assay (ELISA) of serum MDK  2-Reverse Transcriptase– Polymerase Chain Reaction of Midkine gene expression for Paired HCC and Adjacent Noncancerous Liver Tissue | 1-Gene expression of midkine had significant difference in HCC than noncancerous hepatic tissues.  2- Serum midkine levels may be used in conjunction with AFP levels to increase the sensitivity of HCC detection.  3- Serum levels of midikine were more likely to be elevated in patients with HCC desipte its non specificity for HCC.  4- Longitudinal measurements of serum midkine levels in the same person could be a more effective way of monitoring HCC disease activity.  5- Rapidly rising serum midkine levels always took place shortly before the death of patients due to HCC progression  6- Serum midkine levels were unable to detect the development of new HCC inpatients in complete remission or the development of de novo HCC in high-risk people |
